# Molecular signature of cell cycle exit induced in human T lymphoblasts by IL-2 withdrawal

**DOI:** 10.1186/1471-2164-10-261

**Published:** 2009-06-08

**Authors:** Magdalena Chechlinska, Jan Konrad Siwicki, Monika Gos, Malgorzata Oczko-Wojciechowska, Michal Jarzab, Aleksandra Pfeifer, Barbara Jarzab, Jan Steffen

**Affiliations:** 1 Department of Immunology, Maria Sklodowska-Curie Memorial Cancer Centre and Institute of Oncology, Warsaw, Poland; 2 Department of Cell Biology, Maria Sklodowska-Curie Memorial Cancer Centre and Institute of Oncology, Warsaw, Poland; 3 Department of Nuclear Medicine and Endocrine Oncology, Maria Sklodowska-Curie Memorial Cancer Centre and Institute of Oncology, Gliwice, Poland; 4 Department of Tumor Biology and Clinical Oncology, Maria Sklodowska-Curie Memorial Cancer Centre and Institute of Oncology, Gliwice, Poland

## Abstract

**Background:**

The molecular mechanisms of cell cycle exit are poorly understood. Studies on lymphocytes at cell cycle exit after growth factor deprivation have predominantly focused on the initiation of apoptosis. We aimed to study gene expression profile of primary and immortalised IL-2-dependent human T cells forced to exit the cell cycle by growth factor withdrawal, before apoptosis could be evidenced.

**Results:**

By the Affymetrix microarrays HG-U133 2.0 Plus, 53 genes were distinguished as differentially expressed before and soon after IL-2 deprivation. Among those, *PIM1, BCL2, IL-8, HBEGF, DUSP6, OSM, CISH, SOCS2, SOCS3, LIF *and *IL13 *were down-regulated and *RPS24, SQSTM1, TMEM1, LRRC8D, ECOP, YY1AP1, C1orf63, ASAH1, SLC25A46 *and *MIA3 *were up-regulated. Genes linked to transcription, cell cycle, cell growth, proliferation and differentiation, cell adhesion, and immune functions were found to be overrepresented within the set of the differentially expressed genes.

**Conclusion:**

Cell cycle exit of the growth factor-deprived T lymphocytes is characterised by a signature of differentially expressed genes. A coordinate repression of a set of genes known to be induced during T cell activation is observed. However, growth arrest following exit from the cell cycle is actively controlled by several up-regulated genes that enforce the non-dividing state. The identification of genes involved in cell cycle exit and quiescence provides new hints for further studies on the molecular mechanisms regulating the non-dividing state of a cell, the mechanisms closely related to cancer development and to many biological processes.

## Background

Cell growth, proliferation and arrest mechanisms are crucial for development and homeostasis. Extensive studies have examined the mechanisms of cell activation and cell cycle progression, which involve sequential effects of growth factors and their receptors and activation of intracellular signal transduction pathways, transcription factors, cyclins and cyclin-dependent kinases [1]. Studies on lymphocytes withdrawn from the cell cycle have focused on the initiation of apoptosis following growth factor deprivation [2-8] or on the cell-to-cell contact mechanisms that prevent cells from apopotosis [9-11]. Still, the molecular mechanisms of cell cycle exit remain poorly understood. With the lack of mitogenic signals, and in response to antiproliferative signals such as mitogen withdrawal, growth factor starvation, contact inhibition or DNA damage, a proliferating cell arrests to become quiescent (a reversible, nondividing state) or senescent, or to undergo apoptotic death. Most somatic cells in an adult body remain in a postmitotic G_0 _phase. However, during tissue regeneration and repair, wound healing and immune response, cells re-enter the cell cycle and subsequently withdraw from proliferation.

Studies in lymphocytes and fibroblasts have shown that resting cells are not in a passive state resulting simply from the lack of stimulation. Pajalunga et al. [12] summarized cell cycle exit as "a shift in the balance between positive and negative regulators of proliferation in favour of the latter". Indeed, resting cells were found to express sets of up-regulated and down-regulated genes that maintained an active state of non-division [1,12,13,15,16]. A group of genes required for cell cycle exit and the maintenance of cell quiescence in human fibroblasts following serum deprivation has been recently identified [17]. Comparative studies of transcriptional profiles of resting and stimulated lymphocytes [1,18] have shown that T lymphocyte quiescence is actively maintained by products of a set of genes, including *TOB *and *KLF *(*LKLF, GKLF *and *BKLF) *and *FOXO *families of transcription factors. Other candidate genes involved in lymphocyte quiescence, including *TSC-22 *and *Dyrk1*, have been identified in a mouse model among genes highly expressed in resting lymphocytes and down-regulated after T or B cell activation [1].

The present study on a human T cell model may provide new insights into the molecular changes involved in cell proliferation control, cell cycle exit and cellular quiescence. Cellular quiescence represents an important safeguard against tumorigenesis; therefore the identification of quiescence-controlling genes and a better understanding of the relevant molecular pathways may open new possibilities in cancer diagnosis and treatment. Thus, in our study, proliferating interleukin (IL)-2-dependent human T cells were forced to exit cell cycle by growth factor withdrawal, and their gene expression profiles were examined.

## Results and discussion

To gain new insights into cell cycle exit of T lymphocytes, we performed gene expression profiling in human IL-2-dependent T lymphoblasts deprived of the growth factor. Following IL-2 withdrawal, IL-2-dependent, proliferating T lymphoblasts cease to divide and undergo apoptosis [19]. Therefore, transcription profiles of T cells were analysed soon after IL-2 withdrawal (8 hours), before apoptotic changes could be observed. In addition, to minimize effects associated with the heterogeneity of primary T cell populations, we also studied clonal populations of immortalised IL-2-dependent T lymphoblasts and looked for the common changes in the two cellular models. We believe that this approach also limited the "non-specific" gene expression changes in the primary IL-2-deprived T lymphoblasts, i.e. not related to IL-2 withdrawal, but rather to the possible paracrine signalling in polyclonal populations by other cytokines characterised by a redundant activity.

Five cell lines were subjected to IL-2 deprivation and microarray analysis, three primary, IL-2-dependent T lymphoblast lines derived from three different donors (denoted "6", "43" and "j") and two immortalised T cell lines (denoted line 5 and S9), examined in three biological replicates each. Altogether, 18 microarray hybridisations were performed.

**Figure 1:**
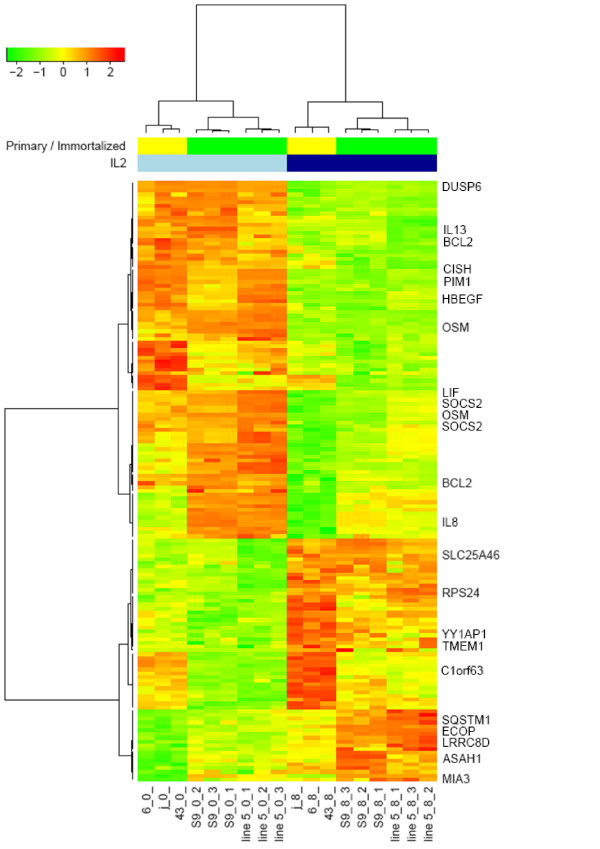
**Hierarchical clustering analysis based on the expression values of 158 probesets differentiating samples before and after IL-2 withdrawal in the non-paired analysis**. Samples before IL-2 withdrawal are in the light-blue columns, samples after IL-2 withdrawal in the navy-blue columns. Primary samples are in the yellow column, immortalised samples are in the green one. The names of the genes of the "T lymphocyte cell cycle exit signature" (Table 2) are marked.

In the first step, unsupervised Principal Component Analysis was performed to assess the reliability of the experiments. Biological replicates were shown to cluster together. The gene expression profile of all the analysed cell lines was shown to be affected by IL-2 withdrawal, but the major variability of the gene expression profile in the analysed dataset was found between primary and immortalised cell lines (unpublished data). Differences between primary and immortalised cells were much greater than those resulting from IL-2 withdrawal. Therefore, our analysis was carried out in a block design, and changes in gene expression following IL-2 withdrawal were assessed separately in primary and immortalised cell lines. By means of the parametric non-corrected t-test we selected 158 differentially expressed genes at p < 0.001 (see Additional File 1: Table A1 for genes differentiating between samples before and after IL-2 withdrawal, selected by a non-paired analysis). The probability of a random selection of such a number of genes was low (0.03 in a global test) which confirmed the reliability of the observed differences. A false discovery rate for the selected genes ranged from 0.003 to 0.12. The hierarchical clustering of the 158 differentially expressed probe-sets obtained in this analysis resulted in an ideal discrimination between the samples before and after IL-2 withdrawal, with a rather uniform pattern of expression (Figure 1).

Having confirmed the statistical significance of differences related to IL-2 withdrawal, we have selected the most consistently changed genes in all five different cell lines analysed, by employing a paired analysis (of the corresponding cells before and after IL-2 withdrawal). The univariate non-corrected permutation test revealed 166 probe-sets changed significantly at P < 0.001 (while in a global test, a borderline significance of P = 0.0625 was obtained). A hundred and eight of these genes were repressed after growth factor withdrawal, and the remaining 58 were up-regulated (see Additional File 2: Table A2 for genes differentiating between samples before and after IL-2 withdrawal, selected by a paired sample analysis). Out of those, *DUSP6, OSM, CISH, SOCS2, LIF *and *IL13 *were found to be the most prominently down-regulated (more than 20-fold), and the expression of *EMB, SOX4, RPS24, WNT5B, SLC1A4, SQSTM1, SUMF1, TMEM1, STK38, LAMP2 *and *C17orf42 *was found to be the most increased (2-fold to 3.5-fold change).

We carried out an external validation of the results obtained in the microarray analysis by a qRT-PCR. For validation we used the same cell lines and we chose 8 genes from the list of 158 most significantly changed transcripts, and a further 4 genes, known for their interesting function, with lower significance of differences. Most of the qRT-PCR results confirmed the microarray findings (Table 1): 7 of 8 top genes and 3 of 4 less significant transcripts were confirmed in a qRT-PCR experiment, usually with a similar fold-change.

**Table 1:** Validation of the selected microarray data by the qRT-PCR.

Rank	Gene symbol	Description	Probe set	Signal Log Ratio (microarray analysis)										Micorarray Fold-change (after/before IL2 withdrawal)	Parametric p-value (non-paired comparison)	qRT-PCR Fold change	qRT-PCR p-value (non-paired)
								
				j		43		6		line 5		S9					
**genes with P < 0.0001 in the microarray gene expression analysis**																	

2	CISH	cytokine inducible SH2-containing protein	223377_x_at	-5.01		-4.936		-5.435		-4.649		-4.519		0.03	<0.0001	0.08	<0.0001
7	DUSP6	dual specificity phosphatase 6	208893_s_at	-6.236		-5.771		-5.75		-4.873		-5.028		0.02	<0.0001	0.04	<0.0001
11	C3orf59	chromosome 3 open reading frame 59	227599_at	-3.035		-2.559		-2.647		-2.401		-2.29		0.17	<0.0001	0.32	0.0082
18	ASAH1	N-acylsphingosine amidohydrolase (acid ceramidase) 1	213702_x_at	0.726		0.671		0.612		0.569		0.787		1.59	<0.0001	2.08	0.0069

**genes with P < 0.001 in the microarray gene expression analysis**																	

33	SOCS2	suppressor of cytokine signaling 2	203373_at	-4.178		-4.954		-5.332		-3.528		-4.438		0.04	0.0001	0.12	0.0058
45	OSM	oncostatin M	214637_at	-4.201		-4.671		-3.352		-4.564		-5.351		0.04	0.0002	0.04	0.0011
66	IL8	interleukin 8	211506_s_at	-3.537		-2.395		-3.14		-4.011		-3.575		0.10	0.0003	0.19	0.2333
92	HBEGF	heparin-binding EGF-like growth factor	203821_at	-5.121		-3.605		-4.72		-3.679		-3.178		0.06	0.0004	0.08	0.0001

**genes with P < 0.01 in the microarray gene expression analysis**																	

221	MT1F	metallothionein 1F	217165_x_at	2.408		1.794		1.091		2.344		2.002		3.80	0.0013	7.05	0.0004
348	MT1X	metallothionein X	208581_x_at	2.066		1.764		0.997		2.656		2.005		3.73	0.0021	4.76	0.0004
514	CLK4	CDC-like kinase 4	210346_s_at	1.399		1.014		1.729		1.188		0.576		2.27	0.0036	2.41	0.0046
600	IL7R	interleukin 7 receptor	205798_at	1.923		0.919		1.965		2.962		1.721		3.73	0.0043	3.19	0.2734

To select transcripts consistently changed upon IL-2 withdrawal, we combined the two lists of 166 and 158 significant genes, obtained in the paired and non-paired analyses, respectively. We identified a set of 53 genes comprised of 13 up-regulated and 40 down-regulated genes, which we designated a "T lymphocyte cell cycle exit signature" (Table 2).

**Table 2:** Gene expression signature of T lymphocyte cell cycle exit.

	**Probe set**	**Gene symbol**	**Description**	**Fold-change (after IL-2 withdrawal vs. before)**	**Paired design**		**Non-paired design**	
					
					**Parametric p-value**	**FDR^1^**	**Parametric p-value**	**FDR**
**Up-regulated**								

1	1555878_at	RPS24	ribosomal protein S24	3.21	0.0003	0.30	<0.0001	0.03
2	201471_s_at	SQSTM1	sequestosome 1	2.48	0.0002	0.30	0.0004	0.09
3	226831_at	NA	NA	2.04	0.0004	0.30	<0.0001	<0.01
4	1555446_s_at	TMEM1	transmembrane protein 1	2.04	<0.0001	0.30	<0.0001	<0.01
5	218684_at	LRRC8D	leucine rich repeat containing 8 family, member D	1.72	0.0007	0.30	0.0003	0.09
6	208091_s_at	ECOP	EGFR-coamplified and overexpressed protein	1.69	0.0006	0.30	<0.0001	0.06
7	244803_at	YY1AP1	YY1 associated protein 1	1.66	0.0002	0.30	0.0004	0.09
8	209006_s_at	C1orf63	chromosome 1 open reading frame 63	1.65	0.0006	0.30	0.0007	0.10
9	213702_x_at	ASAH1	N-acylsphingosine amidohydrolase (acid ceramidase) 1	1.59	<0.0001	0.30	<0.0001	0.02
10	212833_at	SLC25A46	solute carrier family 25, member 46	1.58	0.0006	0.30	0.0005	0.10
11	212310_at	MIA3	melanoma inhibitory activity family, member 3	1.50	0.0007	0.30	<0.0001	0.03
12	1562612_at	ME2	malic enzyme 2, NAD(+)-dependent, mitochondrial	1.47	<0.0001	0.309	<0.0001	<0.01
13	230161_at	CD99	CD99 molecule	1.45	0.0005	0.30	<0.0001	<0.01

**Down-regulated**								

1	1553101_a_at	ALKBH5	alkB, alkylation repair homolog 5 (E. coli)	0.72	0.0003	0.23	0.0009	0.11
2	201968_s_at	PGM1	phosphoglucomutase 1	0.64	0.0006	0.22	0.0006	0.10
3	206055_s_at	SNRPA1	small nuclear ribonucleoprotein polypeptide	0.62	0.0005	0.22	0.0005	0.10
4	228146_at	C17orf51	chromosome 17 open reading frame 51	0.55	0.0007	0.22	0.0007	0.10
5	207339_s_at	LTB	lymphotoxin beta (TNF superfamily, member 3)	0.54	0.0002	0.22	0.0008	0.11
6	201170_s_at	BHLHB2	basic helix-loop-helix domain containing, class B, 2	0.53	<0.0001	0.22	<0.0001	0.04
7	202932_at	YES1	v-yes-1 Yamaguchi sarcoma viral oncogene homolog 1	0.50	0.0008	0.224	0.0009	0.11
8	206999_at	IL12RB2	interleukin 12 receptor, beta 2	0.49	0.0002	0.22	0.0005	0.10
9	201041_s_at	DUSP1	dual specificity phosphatase 1	0.44	0.0005	0.21	<0.0001	0.05
10	202081_at	IER2	immediate early response 2	0.43	0.0005	0.21	0.0009	0.11
11	227262_at	HAPLN3	hyaluronan and proteoglycan link protein 3	0.41	0.0005	0.20	0.0006	0.10
12	218551_at	RP5-1077B9.4	invasion inhibitory protein 45	0.41	0.0004	0.20	0.0006	0.10
13	212590_at	RRAS2	related RAS viral (r-ras) oncogene homolog 2	0.40	0.0002	0.20	0.0002	0.07
14	226283_at	WDR51B	WD repeat domain 51B	0.39	0.0004	0.20	<0.0001	0.01
15	219681_s_at	RAB11FIP1	RAB11 family interacting protein 1 (class I)	0.38	0.0004	0.20	<0.0001	0.03
16	219255_x_at	IL17RB	interleukin 17 receptor B	0.38	0.0004	0.20	0.0005	0.10
17	224156_x_at	IL17RB	interleukin 17 receptor B	0.37	0.0007	0.20	0.0006	0.10
18	210845_s_at	PLAUR	plasminogen activator, urokinase receptor	0.36	0.0004	0.20	0.0003	0.08
19	214508_x_at	CREM	cAMP responsive element modulator	0.30	0.0007	0.20	<0.0001	0.05
20	209488_s_at	RBPMS	RNA binding protein with multiple splicing	0.28	0.0007	0.20	<0.0001	0.05
21	223217_s_at	NFKBIZ	nuclear factor of kappa light polypeptide gene enhancer in B-cells inhibitor, zeta	0.25	0.0008	0.20	0.0005	0.10
22	209193_at	PIM1	pim-1 oncogene	0.20	<0.0001	0.20	<0.0001	<0.01
23	225262_at	FOSL2	FOS-like antigen 2	0.17	0.0004	0.20	0.0006	0.10
24	227599_at	C3orf59	chromosome 3 open reading frame 59	0.17	<0.0001	0.19	<0.0001	<0.01
25	244035_at	BCL2	B-cell CLL/lymphoma 2	0.16	0.0008	0.19	0.0009	0.11
26	202859_x_at	IL8	interleukin 8	0.15	0.0009	0.19	0.0006	0.10
27	211506_s_at	IL8	interleukin 8	0.10	0.0003	0.19	0.0002	0.07
28	203821_at	HBEGF	heparin-binding EGF-like growth factor	0.06	0.0004	0.19	0.0002	0.07
29	207844_at	IL13	interleukin 13	0.05	<0.0001	0.19	0.0007	0.10
30	214637_at	OSM	oncostatin M	0.05	0.0002	0.18	<0.0001	<0.01
31	203373_at	SOCS2	suppressor of cytokine signaling 2	0.04	0.0001	0.17	<0.0001	0.03
32	205266_at	LIF	leukemia inhibitory factor (cholinergic differentiation factor)	0.04	0.0003	0.17	<0.0001	0.03
33	203372_s_at	SOCS2	suppressor of cytokine signaling 2	0.03	0.0002	0.17	<0.0001	0.03
34	208891_at	DUSP6	dual specificity phosphatase 6	0.03	0.0002	0.17	<0.0001	<0.01
35	223377_x_at	CISH	cytokine inducible SH2-containing protein	0.03	<0.0001	0.16	<0.0001	<0.01
36	223961_s_at	CISH	cytokine inducible SH2-containing protein	0.03	<0.0001	0.14	<0.0001	0.01
37	221223_x_at	CISH	cytokine inducible SH2-containing protein	0.03	<0.0001	0.13	<0.0001	<0.01
38	208893_s_at	DUSP6	dual specificity phosphatase 6	0.02	<0.0001	0.13	<0.0001	<0.01
39	230170_at	OSM	oncostatin M	0.02	0.0002	0.13	<0.0001	<0.01
40	208892_s_at	DUSP6	dual specificity phosphatase 6	0.02	0.0010	0.05	<0.0001	0.03

Common genes identified as significantly changed (p < 0.001) in a paired analysis and in a non-paired block analysis.

^1^FDR – False Discovery Rate

The up-regulated genes identified after IL-2 deprivation in both primary and immortalised cells included *SQSTM1, ECOP, YY1AP1, RPS24, TMEM1, LRRC8D, C1orf63, ASAH1, SLC25A46 *and *MIA3*, some of which have been associated with cellular growth control.

The SQSTM1 protein, previously known as p62 protein, interacts with the ubiquitinated proteins to mediate their clearance and is an important scaffold molecule in the RANK-NF-kappaB signalling pathway [20]. In addition, while the p62 protein is also a ligand for Lck kinase of the Src protein-tyrosine kinase family that is involved in T-cell receptor-dependent activation [21], Köller et al. [22] stress the role of Lck in transient T cell unresponsiveness, mediated by a selective IL-2 deficiency. Thus, SQSTM1 may play an important role in cell growth, especially because it may facilitate cell survival through signalling cascades for example those that result in Akt activation, by interaction with protein kinase C, as shown in human neuronal cells [23].

Interestingly, the product of the *ECOP *gene, which was found here to be up-regulated, has also been previously shown to be involved in NF-kappa B-related regulation of cellular growth. ECOP (EGFR-Co-amplified and over-expressed protein) has been implicated as a key regulator in the NF-kappaB signalling, and it has been postulated that high-level, amplification-mediated *ECOP *expression, such as that occurring in tumours with amplified EGFR, could contribute to resistance to apoptosis [24].

The identified genes may as well be associated with other cell proliferation pathways. For example, a product of the *YYAP1 *gene, called YY1-associated protein, a co-activator of the YY1 (Yin Yang 1) transcription factor (YYAP1), had been initially identified as a long splice variant of HCC (hepatocellular carcinoma)-specific protein encoded by the *HCCA2 *gene (HCC-associated gene 2). An over-expression of *HCCA2 *has been found to lead to a cell cycle arrest at G0/G1 phase and to an inhibition of cell proliferation [25].

For the other up-regulated genes distinguished in this study, we could not identify links to the known mechanisms of cell cycle arrest, but some of the genes have been previously associated with different cancers. An over-expression of *ASAH1 *has been found in different malignant tumours, including prostate cancer, head and neck squamous cell cancers and T-cell large granular lymphocyte (LGL) leukaemia [26-28]. An inhibition of acid ceramidase, the product of *ASAH1*, the over-expression of which in cancer cells has been implicated in drug resistance, is suggested as an efficient and promising novel treatment strategy [29,30]. The ribosomal protein gene *RPS24 *has been associated with hepatocellular carcinoma [14]. The MIA3 (TANGO) protein has been identified as a tumour suppressor in malignant melanoma and in colon and hepatocellular carcinomas [31,32].

Out of genes encoding for the suggested crucial regulators of T lymphocyte quiescence, such as TOB and members of the KLF and FOXO transcription factors' families, as well as TSC-22 and Dyrk1, which had previously been found to be overexpressed in resting T cells [1,18,33-35], we only found the *FOXO3 *gene to be slightly, but significantly up-regulated after IL-2 withdrawal. This discrepancy might be explained by differences between the different cell models employed (mouse vs. human) and by different experimental settings. On the other hand, the activity of these transcription factors may be regulated posttranscriptionally, so that their mRNA levels may not necessarily reflect their activity. Thus, the expression of some of the known FOXO target genes was examined and found to be affected following IL-2 withdrawal. *IL7R*, the FOXO1 target gene was up to 4-fold overexpressed in the primary T lymphocytes, but not in immortalised T cells. *CDKN1B *(*p27)*, another FOXO target in human, mouse and C. elegans models [1,34-37], encoding a cell-cycle regulatory protein, presented slightly, but significantly increased expression. Similarly, *Pink1*, a recently identified FOXO target, the product of which helps to protect lymphocytes from apoptosis after growth factor deprivation [38], was among genes of significantly elevated expression in our model of cell-cycle exit. Studies of Murphy [36] on C. elegans model pointed at metallothionein genes as possible FOXO targets. In the present study, several metallothionein genes (*MT1H*, *MT1X*, *MT1G*, *MT2A*, *MT1M*, *MT1E*, *MT1F*) were overexpressed (2.6 to 3.8 times) following IL-2 withdrawal. In addition, we show here, that *Cyclin D *expression, reported to be downregulated by FOXO and KLF [1,35], decreased after IL-2 withdrawal. However, some genes reported by other research groups as human FOXO targets, such as *p130 *gene, *Cyclin G2 *[37], or *IGF1R*, *BCL-2BP*, or *Sestrin 1 *found to be FOXO3a-inducible genes in mouse T cells [34], as well as most of the KLF targets summarised by Tsachanis [16] and Yusuf [33], were unaffected by IL-2 deprivation in our experimental model.

A set of genes identified as significantly down-regulated 8 hours following IL-2 withdrawal included *PIM1, BCL2, IL-8, HBEGF, DUSP6, OSM, CISH, SOCS2, LIF *and *IL13*. It is notable that some of the identified genes, namely *PIM1, BCL2, DUSP6, CISH and SOCS2 *were previously demonstrated to be the IL-2 target genes or the regulators of IL-2 signalling [39-41]. In addition, it is clear that *PIM1, CISH *and suppressors of cytokine signalling (*SOCS*) are implicated in the biological actions of IL-2 [42], and that IL-2 was found to regulate mRNA levels of *SOCS2, CISH, DUSP5 *and *DUSP6 *[41]. It has also been demonstrated that OSM can quickly up-regulate *CISH *[43]. The coordinated repression of *OSM *and *CISH *that we showed in T cells following IL-2 withdrawal is consistent with these observations.

To perform a functional analysis of the up- and down-regulated genes, the 158 genes selected in the non-paired comparison between IL-2-deprived and non-deprived lymphocytes were first analysed by Gene Ontology (GO) Categories (see Additional File 3. Table A3 for gene ontology analysis of genes selected in a non-paired sample comparison between IL-2-deprived and non-deprived lymphocytes). The number of genes changed in each category was compared with the expected quantity of genes and the greatest enrichment was found in two molecular function (MF) classes, GO:0016868, intramolecular transferase activity, phosphotransferases; (observed/expected ratio = 43.9) and GO:0005126, hematopoietin/interferon-class (D200-domain) cytokine receptor binding, (observed/expected ratio = 26.3) and two biological process (BP) classes, GO:0007259, JAK-STAT cascade (observed/expected ratio = 16.7) and GO:0009968, negative regulation of signal transduction, (observed/expected ratio = 6.8). Secondly, a gene set analysis was performed by comparing the overall significance of gene groups defined by GO categories. Out of the 2348 GO classes 317 were found to be significantly affected by IL-2 withdrawal, as shown by at least one of the three tests used to assess the significance of the differences. The selected GO categories are shown in Table 3. The LS/KS permutation tests, which find gene sets that have more differentially expressed genes among the classes than expected by chance, identified 206 significant gene-sets. Efron-Tibshirani's test, which uses 'maxmean' statistics to identify differentially expressed gene-sets, found 231 significant gene-sets (under 200 permutations). Eleven out of the 215 cellular component categories, 69 of 545 molecular function (MF) categories and 237 of 1588 biological process (BP) categories were significant. Thirty-nine GO categories with p < 0.005 in all of the three applied gene group significance tests were identified (see Additional File 4: Table A4 for overall significance of expression changes following IL-2 deprivation in gene groups defined by GO categories).

**Table 3:** Selected Gene Ontology GO categories significantly affected by genes deregulated following IL-2 deprivation.

**GO category**	**GO categories**	**GO term**	**LS permutation p-value**	**KS permutation p-value**	**Efron-Tibshirani's GSA test** **p-value**	**Up-regulated genes** **(p < 0.01)**	**Down-regulated genes** **(p < 0.01)**
GO:0004907	MF	interleukin receptor activity	0.00001	0.00001	< 0.005	*IL12RB1, IL7R*	*IL2RA, IL12RB2, IL17RB IL1RAP*
GO:0019955	MF	cytokine binding	0.00001	0.00001	< 0.005	*IL12RB1, IL7R*	*IL2RA, IL12RB2, IL17RB IL1RAP*
GO:0004896	MF	hematopoietin/interferon-class (D200-domain) cytokine receptor activity	0.00001	0.0000988	< 0.005	*IL12RB1, IL7R*	*IL2RA, IL12RB2, IL17RB IL1RAP*
GO:0007259	BP	JAK-STAT cascade	0.00001	0.0001056	< 0.005		*SOCS1, SOCS2, SOCS3, OSM, STAT4*
GO:0006959	BP	humoral immune response	0.00001	0.0006364	< 0.005		*BCL2, CD55, CFHR1, TNF, LTA, CFH, BCL3, GPI*
GO:0050900	BP	leukocyte migration	0.00001	0.0047229	< 0.005	*MYH9*	*IL8, TNF, CKLF, ITGB2*
GO:0017040	MF	ceramidase activity	0.00001	0.0049829	< 0.005	*ASAH1*	
GO:0018108	BP	peptidyl-tyrosine phosphorylation	0.0001871	0.0032075	< 0.005	*CLK4*	*OSM, SOCS1, IL5, ITGB2*
GO:0006672	BP	ceramide metabolic process	0.0004208	0.0030228	< 0.005	*ASAH1*	*UGCG, CLN8*
GO:0007260	BP	tyrosine phosphorylation of STAT protein	0.0004601	0.0046325	< 0.005		*OSM, SOCS1*
GO:0030218	BP	erythrocyte differentiation	0.0013289	0.00001	< 0.005	*FOXO3, BCL6*	*SFXN1*
GO:0042379	MF	chemokine receptor binding	0.001562	0.0008411	< 0.005		*IL8, CCL3, CKLF*
GO:0048660	BP	regulation of smooth muscle cell proliferation	0.003213	0.0021998	< 0.005		*HBEGF*

Three independent tests: LS, KS and Efron-Tibshirani GSA were applied to select significantly affected gene classes.

The same analytical procedure applied to the database of biological pathways (Biocarta, human database) resulted in 54 significantly up-regulated gene sets. Selected pathways affected by IL-2 withdrawal are shown in Table 4. Both analyses, GO and Biocarta, confirmed the consistent up-regulation of the genes belonging to the JAK-STAT pathway.

**Table 4:** A selection of significantly impacted pathways (BioCarta) in T lymphocytes following IL-2 deprivation.

**Pathway description**	**Biocarta Pathway**	**LS permutation** **p-value**	**KS permutation** **p-value**	**Efron-Tibshirani's GSA test p-value**	**Up-regulated genes ** **(p < 0.01)**	**Down-regulated genes** **(p < 0.01)**
Visceral Fat Deposits and the Metabolic Syndrome	h_vobesityPathway	0.00001	0.0001273	< 0.005	*NR3C1*	*TNF, HSD11B1*
Cells and Molecules involved in local acute inflammatory response	h_LairPathway	0.00001	0.0007952	< 0.005	*ITGB1, ITGAL*	*IL8, TNF, ICAM1, ITGA4*
Adhesion and Diapedesis of Lymphocytes	h_lympathway	0.00001	0.0029985	< 0.005	*ITGB1, ITGAL*	*IL8, ICAM1, ICAM2, ITGA4*
Adhesion and Diapedesis of Granulocytes	h_granulocytesPathway	0.00001	0.0032844	< 0.005		*IL-8, TNF, CD54, ICAM2*
Cytokine Network	h_cytokinePathway	0.00001	0.0297027	< 0.005		*IL13, IL8, LTA, IL-5*
IL-2 Receptor Beta Chain in T cell Activation	h_il2rbPathway	0.00001	0.030315	< 0.005		*CISH, IL2RA, SOCS3, BCL2, AKT1, JAK3*
Induction of apoptosis through DR3 and DR4/5 Death Receptors	h_deathPathway	0.00001	0.00001	0.06		*BCL2, TRADD*
IL22 Soluble Receptor Signaling Pathway	h_il22bppathway	0.0000955	0.00001	< 0.005		*SOCS3, JAK3*
Selective expression of chemokine receptors during T-cell polarization	h_nktPathway	0.0000994	0.0000936	0.03	*IL12RB1, CCR4*	*ILRB2, CCL3, IL5*
Cytokines and Inflammatory Response	h_inflamPathway	0.0001717	0.078942	< 0.005		*IL13, IL8, TNF, LTA, IL5*
NO2-dependent IL 12 Pathway in NK cells	h_no2il12Pathway	0.0006013	0.0135205	< 0.005	*IL12RB1, CD2*	*IL12RB2*
Regulation of MAP Kinase Pathways Through Dual Specificity Phosphatases	h_dspPathway	0.0007988	0.1717258	< 0.005		*DUSP6, DUSP1*
PTEN dependent cell cycle arrest and apoptosis	h_ptenPathway	0.0015749	0.0100088	< 0.005	*FOXO3, ITGB1, MAPK1*	*AKT1, PDK1, SOS1*
Ceramide Signaling Pathway	h_ceramidePathway	0.0016286	0.005673	< 0.005	*MAPK1*	*BCL2, TRADD*
Th1/Th2 Differentiation	h_th1th2Pathway	0.001734	0.0676275	0.07	*IL12RB1*	*IL2RA, IL12RB2*
Dendritic cells in regulating TH1 and TH2 Development	h_dcPathway	0.0023595	0.0493208	< 0.005		
p53 Signaling Pathway	h_p53Pathway	0.0026524	0.1802796	< 0.005		*BCL2, CDK2, CDKN1A*
Telomeres, Telomerase, Cellular Aging, and Immortality	h_telPathway	0.0367325	0.3499152	< 0.005		*BCL2, AKT1*
Phospholipids as signalling intermediaries	h_edg1Pathway	0.0404929	0.1894782	< 0.005	*ASAH1*	*AKT1*

Testing of 288 Biocarta human pathways identified 54 out of 288 investigated gene-sets that passed the 0.005 significance threshold in at least one test (38 gene-sets in LS/KS test and 36 in Efron-Tibshirani's test).

Our data demonstrate for the first time that IL-2 withdrawal induces a coordinate repression of the same set of genes that have been found to be induced during T cell activation as IL-2 targets [44,45]. A similar "symmetry" has been previously shown in human fibroblasts, where approximately half of the genes of the early response to serum stimulation were correspondingly repressed at the cell cycle exit after mitogen deprivation [17].

A few previous studies have shown that some mRNAs that are expressed in resting T and B lymphocytes become repressed following cell activation, and it was suggested that cell quiescence is under an active transcriptional control [1]. Similarly, as shown recently, exit from the cell cycle of human fibroblasts is under control of a "quiescence program" dependent upon a set of genes that enforce the non-dividing state, and ensure the reversibility of the cell cycle arrest [15,17]. Our data support the idea of cell cycle arrest as an active state, controlled by some up-regulated genes.

While in vitro T lymphocytes deprived of a growth factor would eventually undergo apoptosis, in vivo a small proportion of antigen-activated cells would exit to G_0 _and become quiescent. Cellular quiescence is thought to be an indispensable state for the maintenance of lymphocyte homeostasis following immune response, and therefore it is an important barrier against tumorigenesis [46-48]. Some autoimmune and chronic inflammatory disorders involving an excessive lymphocyte proliferation, were found to be associated with increased risks of lymphoma [44,45]. Thus, it is very likely that a deregulated expression of genes responsible for cellular quiescence may contribute to the development of some lymphoid malignancies.

To assess whether T cells adopt quiescent, memory cell phenotype upon IL-2 withdrawal in our model, we compared the gene expression patterns obtained with those described by other researchers as characterising memory vs. naïve vs. effector T cells. Two molecules known to facilitate T cell homing to lymphoid tissues, SELL (CD62L) and CCR7 are expressed predominantly by naïve and memory T cells [49-52], but *CCR7 *expression seems not to be affected by growth factor withdrawal [52]. In our model of cell cycle exit the *SELL *gene was up-regulated in primary T cell lines, while *CCR7*, was unchanged in primary T cells and slightly down-regulated in immortalised T cells after IL-2 deprivation. Out of other genes identified by Holmes et al. [49] as up-regulated in memory T cells, T cells deprived of IL-2 overexpressed *SMAD3*, *TRIM66 *and *NAP1L3*. There was also an increased expression of *IL7R*, thought to be expressed in memory [53] and naïve [50,52] T cells, but this was found not significant by the qRT-PCR (Table 1) when analysed for all T cells, possibly because this was truth only in primary T cells. The *CD58 *(*LFA3*) gene, encoding an adhesion molecule known to be expressed on activated and memory T cells [54], was indeed highly expressed in actively growing cells and consistently up-regulated after IL-2 withdrawal in our experimental system.

Holmes et al. [49] reported *Granzyme B *and *KLRD1 *expression to be induced in activated T cells. Our experiments showed that growth factor-deprived T cells presented decreased *Granzyme B *expression, while *KLRD1 *expression was slightly upregulated, but only in primary T cells. A hierarchical clustering of some genes outlined by Holmes et al. [49] is shown in an Additional file 5: Figure A1.

Haining et al. [55] demonstrated a gene expression profile common for CD8 and CD4 memory T cells. We showed that some of the genes of this profile were significantly changed at cell cycle exit; there was an increased expression of *S100A4*, *S100A11*, *ANXA1*, *ANXA2*, *CRIP1 *and *CASP1*, which, according to Haining et al. [55], are up-regulated in memory T cells, and a reduced expression of *Granzyme A*, downregulated in memory cells [55].

In summary, T cells withdrawn from cell cycle gained some crucial characteristics of memory T cells. Nevertheless, the cell cycle exit signature that we describe here is different from memory T cell gene expression signatures published so far.

Further studies will reveal whether there is a common mechanism of quiescence for different cell types, such as the "common quiescence program" in fibroblasts stimulated to exit to G_0 _by different stimuli [15]. Interestingly, it has been found recently that a group of genes required for cell cycle exit in human fibroblasts following serum deprivation, were coordinately repressed in many types of human cancers, and repression of these genes predicted an increased risk of cancer progression and death in breast cancer patients [17]. Our studies on a model of human primary and immortalised IL-2-dependent T lymphoblasts extended these observations and brought new insights into the understanding of molecular events important in cell cycle exit, deregulation of which may relate to cancer development.

## Conclusion

Cell cycle exit of growth factor-deprived primary and immortalised T lymphocytes is characterised by a gene expression signature, comprising 13 up-regulated and 40 down-regulated genes. Some of these genes are implicated in the mechanisms of cell growth control and show deregulated expression in different tumours. The gene set identified in the non-paired analysis was enriched in transcription, cell cycle, cell growth, proliferation and differentiation, cell adhesion, and immune function genes. Cells at cell cycle exit seem to be under an active transcriptional control. Upon IL-2 withdrawal, some genes become repressed, including those known to be induced during T-cell activation, while others become up-regulated. The identification of genes involved in cell cycle exit and quiescence provides new hints for further studies on the molecular mechanisms regulating the non-dividing state of a cell, the mechanisms closely related to cancer development and to many biological processes.

## Methods

### Cell sources and cell sample preparation

Primary, IL-2-dependent T lymphoblast cell lines ("6", "43" and "j") were generated and propagated as previously described [56]. Briefly, peripheral blood mononuclear cells (PBMC) derived from three healthy donors ("6", "43" and "j") were suspended in a standard medium (RPMI 1640, 10–12% FCS, 50 μg/ml gentamycin [Sigma]), activated for 24 h with 20 μg/ml of wheat germ agglutinin (Pharmacia) and subsequently cultured in the standard medium supplemented with 20 U/ml of recombinant (r) IL-2 (R&D). Flow cytometry cell surface analysis showed that the three primary T lymphoblast cell populations were composed of T cells exclusively, as they expressed all the pan-T markers, and a subset of the cells also expressed CD4 or CD8 surface molecules (unpublished data).

Viable cells were assessed every 3–7 days by the trypan blue exclusion test and resuspended to the concentration of 0.5–2.0 × 10^5 ^cells/ml in a fresh standard medium supplemented with rIL-2. The number of population doublings (PD) was calculated according to the formula:



The cells "6", "43" and "j" were collected after 4–5, 7–8 and 6–7 PD, respectively. The samples of the three primary cell lines were analysed as biological replicates.

Spontaneously immortalised IL-2-dependent T cell lines were derived from normal spleen (line 5) and from PBMC derived from a Nijmegen Breakage Syndrome patient (line S9), as previously described [56,57]. Samples of the immortalised cell lines were collected in three biological replicates each.

The differential gene expression analysis was performed in cell samples collected directly from the IL-2-containing cultures (samples: 6/0, 43/0, j/0, line 5/0 and S9/0) and after 8-hrs incubation of the lymphoblasts in a standard medium without IL-2, following triple thorough washing of the cells in a standard medium (samples: 6/8, 43/8, j/8, line 5/8 and S9/8). In the cultures deprived of IL-2 for 8 hours, no apoptosis was observed in the primary T lymphoblast lines, and in the immortalised cell lines a negligible proportion of cells had the characteristics of early apoptotic cells, as detected by flow cytometry following Annexin-V staining with the use of the Annexin-V-FLUOS Staining Kit of ROCHE (Table 5). But S-phase depletion and G1/S block were observed, as evaluated by flow cytometry with the use of the BD Cycletest Plus (Becton Dickinson) (unpublished data).

**Table 5:** Percentages of apoptotic and necrotic cells in cultures of IL-2-dependent primary (j, 43) and immortalised (line 5, S9) T lymphoblasts (0 h) and at different time points after IL-2 withdrawal (2 h, 8 h, 24 h), analysed by flow cytometry following Annexin-V staining.

Cell lines	0 h^1^		2 h		8 h		24 h	
	
	A^2^	N^3^	A	N	A	N	A	N
j	0.2^4^	2.1	0.9	1.1	0.4	2.2	0.5	2.0
43	0.5	3.0	0.7	2.0	0.4	2.2	0.7	3.0
line 5	2.3	4.0	3.8	2.3	4.6	2.0	8.1	3.8
S9	5.4	1.3	6.7	1.3	8.4	1.5	6.9	1.1

^1^hours after IL-2 withdrawal

^2^A – apoptosis

^3^N – necrosis

^4^percentage; mean of two replicates

### Microarray procedures

Total RNA was isolated using Nucleospin RNA II kit (Macherey-Nagel, Germany). RNA quality was assessed using the Agilent 2100 Bioanalyzer and RNA 6000 Nano Chip Kit (Agilent Technologies).

The Affymetrix GeneChip Human Genome U133 Plus 2.0 arrays, which measure the expression level of over 47,000 transcripts and variants, including 38,500 well-characterised human genes, were used. cDNA synthesis was carried out from 5 μg of RNA (One-Cycle cDNA Synthesis Kit, Affymetrix). After purification (GeneChip Sample Cleanup Module), 7 μl of double-stranded cDNA were used for biotinylated cRNA synthesis (IVT Labeling Kit, Affymetrix). Labeled cRNA was purified using a GeneChip Sample Cleanup Module, fragmented and hybridised with genome array. Washing, staining with streptavidin-phycoerythrin conjugate and scanning of the arrays in Affymetrx GeneChip 3000 scanner were performed as recommended by the Affymetrix Gene Expression Analysis Technical Manual.

### Data analysis

All 18 arrays were normalised by the GC-RMA algorithm using a Bioconductor gcrma package. We filtered out all genes with small amplitude of differences (less than 10% of samples with at least 1.5-fold difference in either direction from the median gene expression among all samples). The filtered dataset comprised of 18770 probe-sets. The full dataset has been deposited in the Gene Expression Omnibus repository (accession no GSE13909).

Differentially expressed genes were identified by a random-variance t-test, with the statistical significance threshold set at p < 0.001. A stringent significance cut-off was used to limit the number of false positive genes obtained (at this threshold no more than 19 false positives are expected in each analysis). A global test of the differences in expression profiles between the classes was also performed by sample permutation. Simultaneously, we assessed a False Discovery Rate for each probeset using the Benjamini-Hochberg method.

To examine the effects of IL-2 withdrawal, samples were compared either according to the type of the cell line (primary/immortalised) in a block design or in a paired sample t-test analysis (for this analysis the replicates of the same sample were averaged).

For cluster analysis, genes identified in the non-paired analysis were used to obtain a heatmap plot with dendrograms, using Ward's hierarchical cluster algorithm (Bioconductor).

Gene Ontology classes for the resulting lists of genes were compared to the number of genes expected, considering the number of genes on the microarray in each category. GO classes and parent classes with at least 5 observations in the selected subset and with an observed vs expected ratio of at least 2 were analysed.

The statistical significance of global gene expression changes in gene ontology categories and BioCarta pathways was assessed by LS/KS permutation tests and the Efron-Tibshirani's GSA maxmean test. All data analyses were performed using the BRB-ArrayTools (developed by Dr. Richard Simon and BRB Array Tools development team) or by Bioconductor packages.

### Real time RT-PCR validation of gene expression

Based on the microarray results, the following genes were selected for validation by the real-time RT-PCR technique: *DUSP6*, *ASAH1*, *HBEGF*, *MT1F*, *MT1X*, *IL7R*, *IL8*, *CLK4*, *C3orf59*, *SOCS2*, *OSM *and *CISH*. Specific primers were designed using Primer Express v.2.0 software (Applied Biosystems). Detailed primer sequences are available on request.

The cDNA was synthesised with the High-Capacity cDNA Reverse Transcription Kit (Applied Biosystems) using 1 μg of total RNA as a template, and Oligo-dT-Primers (Applied Biosystems), according to the manufacturer's instructions. The real-time (= quantitative, q) RT-PCR reactions were performed with Power SYBR Green PCR Master Mix (Applied Biosystems) using the 7000 Real-Time PCR system (Applied Biosystems). Reaction mixtures (25 μl) were subjected to a two-step program: 95°C for 10 minutes followed by 40 cycles of 95°C for 15 s and 60°C for 1 minute. The data were analysed using ABI PRISM 7000 SDS Software. The relative fold difference in gene expression was calculated by the ΔΔCt method using the *ACTB *gene as a normalisation control. The statistical significance of the fold change was assessed by the ANOVA test (Statistica package).

## Abbreviations

BP: biological process; GO: gene ontology; IL: interleukin; MF: molecular function; PBMC: peripheral blood mononuclear cells; r: recombinant.

## Authors' contributions

MCh conceived of the study and participated in its design and coordination, carried out RNA isolation, analysed data and interpreted the study, drafted, wrote and edited the manuscript. JKS conceived of the study and participated in its design and coordination, carried out cell cultures, analysed data and interpreted the study, drafted and wrote the manuscript. MG carried out real-time PCR verification of gene expression, participated in drafting the manuscript. MO-W performed microarray gene expression profiling. MJ performed the biostatistical analyses, participated in drafting the manuscript. AP participated in the biostatistical analyses. BJ participated in the design of the study. JS conceived of the study and participated in its design and coordination. All authors read and approved the final manuscript.

## Supplementary Material

Additional File 1 **Table A1. Genes differentiating between samples before and after IL-2 withdrawal identified by a non-paired sample T-test**. Genes selected at the nominal univariate comparison level of 0.001. Click here for file

Additional File 2 **Table A2. Genes differentiating between samples before and after IL-2 withdrawal identified by a paired sample T-test**. Genes selected at the nominal univariate comparison level of 0.001. Click here for file

Additional File 3 **Table A3. Gene Ontology (GO) analysis of genes selected in a non-paired sample comparison between IL-2-deprived and non-deprived lymphocytes**. The number of genes changed in each category was compared with the number of expected occurrences. Only GO classes and parent classes with at least 5 observations in the selected subset and with an "observed vs. expected" ratio of at least 2 were shown. Click here for file

Additional File 4 **Table A4. Assessment of overall significance of expression changes following IL-2 deprivation in gene groups defined by Gene Ontology categories**. Three independent tests: LS, KS and Efron-Tibshirani GSA were applied to select significantly affected gene classes. Click here for file

Additional File 5 **Figure A1. Hierarchical clustering analysis of selected memory T cell genes**. The analysis based on the expression values of some of the genes identified by Holmes et. al [49] to characterise memory T cells. Samples before IL-2 withdrawal are in the light-blue columns, samples after IL-2 withdrawal in the navy-blue columns. Primary samples are in the yellow column, immortalised samples are in the green one. Click here for file
