# Supplementary material for: Molecular signature of cell cycle exit induced in human T lymphoblasts by IL-2 withdrawal
Source: BMC Genomics. 2009 Jun 8;10:261. doi: 10.1186/1471-2164-10-261 (PMC2706892; doi:10.1186/1471-2164-10-261)
Supplement: Additional File 1 — Table A1. Genes differentiating between samples before and after IL-2 withdrawal identified by a non-paired sample T-test. Genes selected at the nominal univariate comparison level of 0.001. [file 1471-2164-10-261-S1.pdf]

Table A1. Genes differentiating between samples before and after IL-2 withdrawal identified by a non-paired sample T-test.

Rank	Probe set	Gene symbol	Gene Name	Fold-change	Parametric p-value	FDR[1]
1	208893_s_at	DUSP6	dual specificity phosphatase 6	0.02	2.00E-07	0.003
2	1562612_at	ME2	malic enzyme 2, NAD(+)-dependent, mitochondrial	1.47	3.00E-07	0.003
3	209193_at	PIM1	pim-1 oncogene	0.20	7.00E-07	0.004
4	214637_at	OSM	oncostatin M	0.05	1.30E-06	0.006
5	1555446_s_at	TMEM1	transmembrane protein 1	2.04	2.00E-06	0.008
6	223377_x_at	CISH	cytokine inducible SH2-containing protein	0.03	2.40E-06	0.008
7	221223_x_at	CISH	cytokine inducible SH2-containing protein	0.04	2.80E-06	0.008
8	230170_at	OSM	oncostatin M	0.02	3.90E-06	0.009
9	230161_at	CD99	CD99 molecule	1.45	4.40E-06	0.009
10	208891_at	DUSP6	dual specificity phosphatase 6	0.03	4.70E-06	0.009
11	227599_at	C3orf59	chromosome 3 open reading frame 59	0.17	5.50E-06	0.009
12	223961_s_at	CISH	cytokine inducible SH2-containing protein	0.03	6.80E-06	0.010
13	226283_at	WDR51B	WD repeat domain 51B	0.39	7.10E-06	0.010
14	213702_x_at	ASAH1	N-acylsphingosine amidohydrolase (acid ceramidase) 1	1.59	1.80E-05	0.024
15	217165_x_at	MT1F	metallothionein 1F	3.80	1.94E-05	0.024
16	205266_at	LIF	leukemia inhibitory factor (cholinergic differentiation factor)	0.03	2.09E-05	0.025
17	203373_at	SOCS2	suppressor of cytokine signaling 2	0.04	2.61E-05	0.029
18	212310_at	MIA3	melanoma inhibitory activity family, member 3	1.50	2.91E-05	0.030
19	207630_s_at	CREM	cAMP responsive element modulator	0.35	3.26E-05	0.031
20	219681_s_at	RAB11FIP1	RAB11 family interacting protein 1 (class I)	0.38	3.42E-05	0.031
21	1555878_at	RPS24	ribosomal protein S24	3.21	3.62E-05	0.031
22	203372_s_at	SOCS2	suppressor of cytokine signaling 2	0.03	3.73E-05	0.031
23	208892_s_at	DUSP6	dual specificity phosphatase 6	0.02	3.86E-05	0.031
24	210001_s_at	SOCS1	suppressor of cytokine signaling 1	0.19	4.58E-05	0.035
25	217047_s_at	FAM13A1	family with sequence similarity 13, member A1	0.41	4.60E-05	0.035
26	201170_s_at	BHLHB2	basic helix-loop-helix domain containing, class B, 2	0.53	4.89E-05	0.035
27	238982_at	DENR	density-regulated protein	2.26	5.25E-05	0.036
28	230511_at	CREM	cAMP responsive element modulator	0.26	7.60E-05	0.048
29	201041_s_at	DUSP1	dual specificity phosphatase 1	0.44	7.77E-05	0.048
30	209488_s_at	RBPMS	RNA binding protein with multiple splicing	0.28	7.78E-05	0.048
31	214508_x_at	CREM	cAMP responsive element modulator	0.30	7.86E-05	0.048
32	208091_s_at	ECOP	EGFR-coamplified and overexpressed protein	1.69	9.50E-05	0.056
33	35254_at	TRAFD1	TRAF-type zinc finger domain containing 1	0.66	9.82E-05	0.056

34	231832_at	GALNT4	UDP-N-acetyl-alpha-D-galactosamine:polypeptide N-acetylgalactosaminyltransferase 4 (GalNAc-T4)	0.47	0.000102	0.056
35	223059_s_at	FAM107B	family with sequence similarity 107, member B	1.87	0.0001038	0.056
36	1557145_at	STK38	serine/threonine kinase 38	3.43	0.0001392	0.071
37	209498_at	CEACAM1	carcinoembryonic antigen-related cell adhesion molecule 1 (biliary glycoprotein)	0.10	0.0001397	0.071
38	201824_at	RNF14	ring finger protein 14	1.35	0.0001451	0.072
39	204881_s_at	UGCG	UDP-glucose ceramide glucosyltransferase	0.46	0.0001543	0.074
40	223915_at	BCOR	BCL6 co-repressor	2.08	0.0001605	0.075
41	211506_s_at	IL8	interleukin 8	0.10	0.00017	0.075
42	212590_at	RRAS2	related RAS viral (r-ras) oncogene homolog 2	0.40	0.0001706	0.075
43	203821_at	HBEGF	heparin-binding EGF-like growth factor	0.06	0.0001732	0.075
44	227583_at	POP4	processing of precursor 4, ribonuclease P/MRP subunit (S. cerevisiae)	2.18	0.0001824	0.075
45	213629_x_at	MT1JP	metallothionein 1J (pseudogene)	5.25	0.0001837	0.075
46	226452_at	PDK1	pyruvate dehydrogenase kinase, isozyme 1	0.39	0.000187	0.075
47	1555419_a_at	ASAH1	N-acylsphingosine amidohydrolase (acid ceramidase) 1	1.81	0.0001878	0.075
48	201694_s_at	EGR1	early growth response 1	0.02	0.0001919	0.075
49	214757_at	KIAA1505	KIAA1505 protein	1.51	0.0001948	0.075
50	212225_at	EIF1	eukaryotic translation initiation factor 1	0.26	0.0002062	0.077
51	207543_s_at	P4HA1	procollagen-proline, 2-oxoglutarate 4-dioxygenase (proline 4-hydroxylase), alpha polypeptide I	0.19	0.0002232	0.080
52	202637_s_at	ICAM1	intercellular adhesion molecule 1 (CD54), human rhinovirus receptor	0.42	0.0002319	0.080
53	213073_at	ZFYVE26	zinc finger, FYVE domain containing 26	1.74	0.0002334	0.080
54	209967_s_at	CREM	cAMP responsive element modulator	0.42	0.0002373	0.080
55	235926_at	ANAPC5	anaphase promoting complex subunit 5	1.65	0.0002381	0.080
56	243819_at	GNG2	guanine nucleotide binding protein (G protein), gamma 2	0.25	0.0002388	0.080
57	48659_at	RP5-1077B9.4	invasion inhibitory protein 45	0.38	0.000247	0.081
58	223038_s_at	FAM60A	family with sequence similarity 60, member A	0.68	0.0002518	0.081
59	211456_x_at	MT1P2	metallothionein 1 pseudogene 2	3.01	0.0002613	0.083
60	225557_at	AXUD1	AXIN1 up-regulated 1	0.32	0.0002659	0.083
61	227404_s_at	EGR1	early growth response 1	0.01	0.0002782	0.085
62	236280_at	NA	NA	1.68	0.0002834	0.085
63	202973_x_at	FAM13A1	family with sequence similarity 13, member A1	0.19	0.0002869	0.085
64	210845_s_at	PLAUR	plasminogen activator, urokinase receptor	0.35	0.0002981	0.085
65	1559993_at	SFXN3	sideroflexin 3	0.71	0.0003039	0.085
66	205227_at	IL1RAP	interleukin 1 receptor accessory protein	0.54	0.0003057	0.085
67	210524_x_at	NA	NA	3.82	0.0003073	0.085

68	201169_s_at	BHLHB2	basic helix-loop-helix domain containing, class B, 2	0.47	0.0003079	0.085
69	218684_at	LRRC8D	leucine rich repeat containing 8 family, member D	1.72	0.0003179	0.086
70	235322_at	NA	NA	1.99	0.0003245	0.087
71	210528_at	MR1	major histocompatibility complex, class I-related	1.47	0.0003341	0.088
72	206461_x_at	MT1H	metallothionein 1H	3.38	0.0003405	0.089
73	209007_s_at	C1orf63	chromosome 1 open reading frame 63	1.69	0.0003458	0.089
74	231830_x_at	RAB11FIP1	RAB11 family interacting protein 1 (class I)	0.64	0.0003544	0.089
75	1565692_at	CDR2	cerebellar degeneration-related protein 2, 62kDa	2.17	0.0003581	0.089
76	204326_x_at	MT1X	metallothionein 1X	4.46	0.0003624	0.089
77	201471_s_at	SQSTM1	sequestosome 1	2.48	0.0003658	0.089
78	244803_at	YY1AP1	YY1 associated protein 1	1.66	0.0003815	0.092
79	1555266_a_at	ASXL2	additional sex combs like 2 (Drosophila)	1.59	0.0003924	0.093
80	223058_at	FAM107B	family with sequence similarity 107, member B	1.91	0.0004304	0.098
81	232614_at	BCL2	B-cell CLL/lymphoma 2	0.15	0.0004309	0.098
82	203143_s_at	KIAA0040	KIAA0040	0.48	0.0004351	0.098
83	226370_at	KLHL15	kelch-like 15 (Drosophila)	0.54	0.0004358	0.098
84	203958_s_at	ZBTB40	zinc finger and BTB domain containing 40	1.65	0.0004402	0.098
85	232210_at	BCL2	B-cell CLL/lymphoma 2	0.09	0.0004529	0.100
86	206055_s_at	SNRPA1	small nuclear ribonucleoprotein polypeptide A@#$%&	0.62	0.0004727	0.101
87	1556715_at	PRPSAP1	phosphoribosyl pyrophosphate synthetase-associated protein 1	1.38	0.000474	0.101
88	227847_at	EPM2AIP1	EPM2A (laforin) interacting protein 1	1.94	0.000476	0.101
89	213328_at	NEK1	NIMA (never in mitosis gene a)-related kinase 1	1.72	0.0004767	0.101
90	219128_at	C2orf42	chromosome 2 open reading frame 42	1.64	0.0004846	0.101
91	229509_at	MFSD8	major facilitator superfamily domain containing 8	1.52	0.0004918	0.101
92	212833_at	SLC25A46	solute carrier family 25, member 46	1.58	0.0004997	0.101
93	231808_at	LOC729082	hypothetical protein LOC729082	0.53	0.000503	0.101
94	228963_at	NA	NA	1.53	0.0005196	0.101
95	38037_at	HBEGF	heparin-binding EGF-like growth factor	0.08	0.0005243	0.101
96	243999_at	SLFN5	schlafen family member 5	1.69	0.0005248	0.101
97	226725_at	SLFN5	schlafen family member 5	1.53	0.0005254	0.101
98	226831_at	NA	NA	2.04	0.0005283	0.101
99	219255_x_at	IL17RB	interleukin 17 receptor B	0.38	0.0005355	0.101
100	208456_s_at	RRAS2	related RAS viral (r-ras) oncogene homolog 2	0.36	0.0005412	0.101
101	223217_s_at	NFKBIZ	nuclear factor of kappa light polypeptide gene enhancer in B-cells inhibitor, zeta	0.25	0.0005433	0.101
102	206999_at	IL12RB2	interleukin 12 receptor, beta 2	0.49	0.0005495	0.101

103	201968_s_at	PGM1	phosphoglucomutase 1	0.65	0.000563	0.102
104	218551_at	RP5-1077B9.4	invasion inhibitory protein 45	0.41	0.0005804	0.102
105	235522_at	CLEC2D	C-type lectin domain family 2, member D	1.80	0.0005972	0.102
106	237747_at	NA	NA	3.20	0.0006008	0.102
107	224156_x_at	IL17RB	interleukin 17 receptor B	0.37	0.000601	0.102
108	202638_s_at	ICAM1	intercellular adhesion molecule 1 (CD54), human rhinovirus receptor	0.46	0.0006065	0.102
109	227262_at	HAPLN3	hyaluronan and proteoglycan link protein 3	0.41	0.0006083	0.102
110	225558_at	GIT2	G protein-coupled receptor kinase interactor 2	1.54	0.000612	0.102
111	212227_x_at	EIF1	eukaryotic translation initiation factor 1	0.56	0.0006162	0.102
112	38069_at	CLCN7	chloride channel 7	1.51	0.0006187	0.102
113	201855_s_at	ASCIZ	ATM/ATR-Substrate Chk2-Interacting Zn2+-finger protein	1.40	0.0006226	0.102
114	225059_at	AGTRAP	angiotensin II receptor-associated protein	0.36	0.0006255	0.102
115	202859_x_at	IL8	interleukin 8	0.15	0.0006309	0.102
116	242877_at	LSM14A	LSM14A, SCD6 homolog A (S. cerevisiae)	1.48	0.0006342	0.102
117	225262_at	FOSL2	FOS-like antigen 2	0.17	0.0006368	0.102
118	241342_at	TMEM65	transmembrane protein 65	0.47	0.0006403	0.102
119	1555736_a_at	AGTRAP	angiotensin II receptor-associated protein	0.31	0.0006502	0.102
120	202021_x_at	EIF1	eukaryotic translation initiation factor 1	0.57	0.0006502	0.102
121	241370_at	LOC286052	hypothetical protein LOC286052	0.41	0.0006652	0.103
122	228146_at	C17orf51	chromosome 17 open reading frame 51	0.55	0.0006708	0.103
123	213836_s_at	WIPI1	WD repeat domain, phosphoinositide interacting 1	0.47	0.0006782	0.103
124	220305_at	MGC3260	hypothetical protein MGC3260	1.52	0.0006852	0.103
125	209006_s_at	C1orf63	chromosome 1 open reading frame 63	1.65	0.0006919	0.103
126	201014_s_at	PAICS	phosphoribosylaminoimidazole carboxylase, phosphoribosylaminoimidazole succinocarboxamide synthetase	0.61	0.0006995	0.103
127	204798_at	MYB	v-myb myeloblastosis viral oncogene homolog (avian)	1.96	0.0007067	0.103
128	209999_x_at	SOCS1	suppressor of cytokine signaling 1	0.14	0.0007122	0.103
129	208581_x_at	MT1X	metallothionein 1X	3.73	0.0007149	0.103
130	207844_at	IL13	interleukin 13	0.05	0.0007159	0.103
131	212760_at	UBR2	ubiquitin protein ligase E3 component n-recognin 2	1.76	0.0007444	0.106
132	216336_x_at	MT1M	metallothionein 1M	2.42	0.0007513	0.106
133	202951_at	STK38	serine/threonine kinase 38	1.65	0.000762	0.106
134	212130_x_at	EIF1	eukaryotic translation initiation factor 1	0.56	0.00077	0.106
135	202822_at	LPP	LIM domain containing preferred translocation partner in lipoma	0.52	0.0007728	0.106
136	238880_at	GTF3A	general transcription factor IIIA	2.05	0.0007764	0.106
137	236172_at	LTB4R	leukotriene B4 receptor	0.46	0.0007825	0.106

138	203502_at	BPGM	2,3-bisphosphoglycerate mutase	0.40	0.0007902	0.106
139	203965_at	USP20	ubiquitin specific peptidase 20	1.46	0.0008024	0.106
140	228611_s_at	NA	NA	1.68	0.0008071	0.106
141	207339_s_at	LTB	lymphotoxin beta (TNF superfamily, member 3)	0.54	0.0008146	0.106
142	238542_at	ULBP2	UL16 binding protein 2	0.43	0.0008155	0.106
143	209711_at	SLC35D1	solute carrier family 35 (UDP-glucuronic acid/UDP-N-acetylgalactosamine dual transporter), member D1	1.49	0.0008223	0.106
144	1553423_a_at	SLFN13	schlafen family member 13	0.32	0.000824	0.106
145	1555827_at	CCNL1	cyclin L1	0.40	0.0008276	0.106
146	212261_at	TNRC15	trinucleotide repeat containing 15	1.61	0.0008291	0.106
147	225251_at	RAB24	RAB24, member RAS oncogene family	0.69	0.0008339	0.106
148	202642_s_at	TRRAP	transformation/transcription domain-associated protein	1.66	0.0008476	0.107
149	209795_at	CD69	CD69 molecule	0.06	0.0008486	0.1067
150	202081_at	IER2	immediate early response 2	0.43	0.0008665	0.108
151	1552423_at	ETV3	ets variant gene 3	0.40	0.0008687	0.108
152	202932_at	YES1	v-yes-1 Yamaguchi sarcoma viral oncogene homolog 1	0.50	0.0009064	0.111
153	231259_s_at	CCND2	cyclin D2	0.29	0.0009079	0.111
154	244035_at	BCL2	B-cell CLL/lymphoma 2	0.16	0.0009198	0.112
155	210868_s_at	ELOVL6	ELOVL family member 6, elongation of long chain fatty acids (FEN1/Elo2, SUR4/Elo3-like, yeast)	0.14	0.0009252	0.112
156	235094_at	TPM4	tropomyosin 4	0.34	0.0009351	0.113
157	1553101_a_at	ALKBH5	alkB, alkylation repair homolog 5 (E. coli)	0.72	0.0009486	0.113
158	201534_s_at	UBL3	ubiquitin-like 3	1.62	0.0009961	0.118

Genes selected at the nominal univariate comparison level of 0.001.

[1]FDR – False Discovery Rate
