# Supplementary material for: Molecular signature of cell cycle exit induced in human T lymphoblasts by IL-2 withdrawal
Source: BMC Genomics. 2009 Jun 8;10:261. doi: 10.1186/1471-2164-10-261 (PMC2706892; doi:10.1186/1471-2164-10-261)
Supplement: Additional File 2 — Table A2. Genes differentiating between samples before and after IL-2 withdrawal identified by a paired sample T-test. Genes selected at the nominal univariate comparison level of 0.001. [file 1471-2164-10-261-S2.pdf]

Table A2. Genes differentiating between samples before and after IL-2 withdrawal identified by a non-paired sample T-test.

**Rank**	**Probe set**	**Gene symbol**	**Gene Name**	**Fold-change**	**Parametric p-value**	**FDR**
1	203414_at	MMD	monocyte to macrophage differentiation-associated	0.36	1.00E-06	0.019
2	223377_x_at	CISH	cytokine inducible SH2-containing protein	0.03	6.60E-06	0.059
3	223474_at	C14orf4	chromosome 14 open reading frame 4	0.47	9.40E-06	0.059
4	211269_s_at	IL2RA	interleukin 2 receptor, alpha	0.20	1.32E-05	0.062
5	227697_at	SOCS3	suppressor of cytokine signaling 3	0.05	1.72E-05	0.065
6	208893_s_at	DUSP6	dual specificity phosphatase 6	0.02	2.63E-05	0.066
7	1555446_s_at	TMEM1	transmembrane protein 1	2.04	2.70E-05	0.066
8	38269_at	PRKD2	protein kinase D2	0.62	2.80E-05	0.066
9	209193_at	PIM1	pim-1 oncogene	0.20	3.17E-05	0.066
10	227599_at	C3orf59	chromosome 3 open reading frame 59	0.17	3.55E-05	0.067
11	221223_x_at	CISH	cytokine inducible SH2-containing protein	0.03	4.20E-05	0.068
12	211375_s_at	ILF3	interleukin enhancer binding factor 3, 90kDa	0.84	4.90E-05	0.068 6
13	227718_at	PURB	purine-rich element binding protein B	0.58	4.97E-05	0.068
14	200894_s_at	FKBP4	FK506 binding protein 4, 59kDa	0.71	5.48E-05	0.068
15	224785_at	FAM100B	family with sequence similarity 100, member B	0.46	5.50E-05	0.068
16	228604_at	NA	NA	1.39	6.14E-05	0.068
17	213702_x_at	ASAH1	N-acylsphingosine amidohydrolase (acid ceramidase) 1	1.59	6.60E-05	0.068
18	223961_s_at	CISH	cytokine inducible SH2-containing protein	0.03	6.62E-05	0.068
19	207844_at	IL13	interleukin 13	0.05	6.86E-05	0.068
20	1562612_at	ME2	malic enzyme 2, NAD(+)-dependent, mitochondrial	1.47	9.01E-05	0.073
21	201170_s_at	BHLHB2	basic helix-loop-helix domain containing, class B, 2	0.53	9.24E-05	0.074
22	229442_at	C18orf54	chromosome 18 open reading frame 54	1.43	9.68E-05	0.074
23	213153_at	SETD1B	SET domain containing 1B	1.65	0.0001027	0.074
24	224963_at	SLC26A2	solute carrier family 26 (sulfate transporter), member 2	1.81	0.0001123	0.074

25	223092_at	ANKH	ankylosis, progressive homolog (mouse)	1.67	0.000114	0.074
26	203685_at	BCL2	B-cell CLL/lymphoma 2	0.16	0.0001192	0.074
27	226905_at	FAM101B	family with sequence similarity 101, member B	0.44	0.000124	0.074
28	230127_at	NA	NA	0.35	0.0001299	0.074
29	236008_at	C9orf73	chromosome 9 open reading frame 73	0.25	0.000131	0.074
30	1555766_a_at	GNG2	guanine nucleotide binding protein (G protein), gamma 2	0.41	0.0001332	0.074
31	203373_at	SOCS2	suppressor of cytokine signaling 2	0.04	0.0001365	0.074
32	224314_s_at	EGLN1	egl nine homolog 1 (C. elegans)	0.43	0.0001386	0.074
33	239328_at	RCSD1	RCSD domain containing 1	1.43	0.0001487	0.074
34	203372_s_at	SOCS2	suppressor of cytokine signaling 2	0.03	0.00015	0.074
35	220252_x_at	CXorf21	chromosome X open reading frame 21	1.85	0.0001504	0.074
36	209017_s_at	LONP1	lon peptidase 1, mitochondrial	0.64	0.0001535	0.074
37	209626_s_at	OSBPL3	oxysterol binding protein-like 3	0.71	0.0001581	0.074
38	218324_s_at	SPATS2	spermatogenesis associated, serine-rich 2	0.71	0.0001629	0.074
39	1559964_at	FLJ38717	FLJ38717 protein	2.00	0.0001656	0.074
40	218224_at	PNMA1	paraneoplastic antigen MA1	1.82	0.000167	0.074
41	229001_at	PPP1R3E	protein phosphatase 1, regulatory (inhibitor) subunit 3E	1.93	0.0001707	0.074
42	202855_s_at	SLC16A3	solute carrier family 16, member 3 (monocarboxylic acid transporter 4)	0.27	0.0001716	0.074
43	214637_at	OSM	oncostatin M	0.05	0.0001724	0.074
44	210976_s_at	PFKM	phosphofructokinase, muscle	0.76	0.0001767	0.074
45	201471_s_at	SQSTM1	sequestosome 1	2.48	0.0001798	0.074
46	224799_at	NDFIP2	Nedd4 family interacting protein 2	0.36	0.0001849	0.074
47	203853_s_at	GAB2	GRB2-associated binding protein 2	0.28	0.000186	0.074
48	219221_at	ZBTB38	zinc finger and BTB domain containing 38	1.48	0.000188	0.074
49	212590_at	RRAS2	related RAS viral (r-ras) oncogene homolog 2	0.40	0.000192	0.074
50	230170_at	OSM	oncostatin M	0.02	0.0001964	0.074
51	203904_x_at	CD82	CD82 molecule	1.44	0.0002114	0.076
52	229194_at	PCGF5	polycomb group ring finger 5	0.61	0.0002116	0.076
53	244803_at	YY1AP1	YY1 associated protein 1	1.66	0.0002194	0.076
54	206999_at	IL12RB2	interleukin 12 receptor, beta 2	0.49	0.000222	0.077

55	201037_at	PFKP	phosphofructokinase, platelet	0.53	0.0002329	0.077
56	207339_s_at	LTB	lymphotoxin beta (TNF superfamily, member 3)	0.54	0.0002407	0.077
57	209457_at	DUSP5	dual specificity phosphatase 5	0.33	0.0002426	0.077
58	208891_at	DUSP6	dual specificity phosphatase 6	0.03	0.0002431	0.077
59	201925_s_at	CD55	CD55 molecule, decay accelerating factor for complement (Cromer blood group)	0.53	0.0002461	0.077
60	213737_x_at	GOLGA8G	golgi autoantigen, golgin subfamily a, 8G	0.50	0.0002509	0.077
61	1553101_a_at	ALKBH5	alkB, alkylation repair homolog 5 (E. coli)	0.72	0.0002511	0.077
62	211506_s_at	IL8	interleukin 8	0.10	0.0002539	0.077
63	204629_at	PARVB	parvin, beta	0.68	0.0002665	0.078
64	226876_at	FAM101B	family with sequence similarity 101, member B	0.47	0.0002679	0.078
65	217916_s_at	FAM49B	family with sequence similarity 49, member B	0.71	0.0002689	0.078
66	1555878_at	RPS24	ribosomal protein S24	3.21	0.0002756	0.078
67	235875_at	SLC1A4	solute carrier family 1 (glutamate/neutral amino acid transporter), member 4	2.55	0.0002781	0.078
68	214487_s_at	RAP2B	RAP2B, member of RAS oncogene family	0.76	0.0002874	0.079
69	226671_at	LAMP2	lysosomal-associated membrane protein 2	2.02	0.0002944	0.080
70	227087_at	NA	NA	0.81	0.0003066	0.080
71	227395_at	NA	NA	1.59	0.0003107	0.080
72	225763_at	RCSD1	RCSD domain containing 1	1.42	0.0003123	0.080
73	204131_s_at	FOXO3	forkhead box O3	1.39	0.0003137	0.080
74	227796_at	LOC643836	similar to Zinc finger protein 62 homolog (Zfp-62) (ZT3)	1.41	0.0003173	0.080
75	208748_s_at	FLOT1	flotillin 1	0.33	0.0003181	0.080
76	205266_at	LIF	leukemia inhibitory factor (cholinergic differentiation factor)	0.04	0.0003243	0.080
77	221029_s_at	WNT5B	wingless-type MMTV integration site family, member 5B	2.97	0.0003366	0.082
78	210891_s_at	GTF2I	general transcription factor II, i	1.37	0.0003513	0.083
79	219681_s_at	RAB11FIP1	RAB11 family interacting protein 1 (class I)	0.38	0.0003519	0.083
80	209467_s_at	MKNK1	MAP kinase interacting serine/threonine kinase 1	0.57	0.0003594	0.083
81	225262_at	FOSL2	FOS-like antigen 2	0.17	0.0003595	0.083

82	226831_at	NA	NA	2.04	0.0003735	0.085
83	218551_at	RP5-1077B9.4	invasion inhibitory protein 45	0.41	0.0003769	0.085
84	203821_at	HBEGF	heparin-binding EGF-like growth factor	0.06	0.0003795	0.085
85	218000_s_at	PHLDA1	pleckstrin homology-like domain, family A, member 1	0.27	0.0003889	0.086
86	210845_s_at	PLAUR	plasminogen activator, urokinase receptor	0.36	0.0003995	0.087
87	226031_at	CCDC132	coiled-coil domain containing 132	1.41	0.0004045	0.087
88	226283_at	WDR51B	WD repeat domain 51B	0.39	0.000407	0.087
89	219255_x_at	IL17RB	interleukin 17 receptor B	0.38	0.0004158	0.087
90	213112_s_at	SQSTM1	sequestosome 1	2.50	0.0004188	0.087
91	210980_s_at	ASAH1	N-acylsphingosine amidohydrolase (acid ceramidase) 1	1.61	0.0004323	0.089
92	217997_at	PHLDA1	pleckstrin homology-like domain, family A, member 1	0.27	0.0004497	0.092
93	213189_at	DKFZp667G2110	hypothetical protein DKFZp667G2110	0.53	0.0004548	0.092
94	205423_at	AP1B1	adaptor-related protein complex 1, beta 1 subunit	0.81	0.0004681	0.093
95	227262_at	HAPLN3	hyaluronan and proteoglycan link protein 3	0.41	0.0004794	0.095
96	214334_x_at	DAZAP2	DAZ associated protein 2	1.26	0.0004975	0.096
97	206055_s_at	SNRPA1	small nuclear ribonucleoprotein polypeptide A@#$%&	0.62	0.0005028	0.096
98	240013_at	NA	NA	0.23	0.0005043	0.096
99	226850_at	SUMF1	sulfatase modifying factor 1	2.11	0.0005074	0.096
100	201041_s_at	DUSP1	dual specificity phosphatase 1	0.44	0.0005101	0.096
101	230161_at	CD99	CD99 molecule	1.45	0.0005177	0.096
102	202081_at	IER2	immediate early response 2	0.43	0.0005259	0.096
103	217996_at	PHLDA1	pleckstrin homology-like domain, family A, member 1	0.25	0.0005261	0.096
104	212217_at	PREPL	prolyl endopeptidase-like	1.31	0.000537	0.097
105	225176_at	LNPEP	leucyl/cystinyl aminopeptidase	1.44	0.0005488	0.098
106	209006_s_at	C1orf63	chromosome 1 open reading frame 63	1.65	0.0005551	0.098
107	212833_at	SLC25A46	solute carrier family 25, member 46	1.58	0.0005657	0.099
108	208091_s_at	ECOP	EGFR-coamplified and overexpressed protein	1.69	0.0005782	0.099
109	228828_at	RNF43	ring finger protein 43	1.86	0.0005841	0.099
110	201968_s_at	PGM1	phosphoglucomutase 1	0.65	0.00059	0.099

111	209340_at	UAP1	UDP-N-acteylglucosamine pyrophosphorylase 1	0.82	0.000592	0.099
112	209390_at	TSC1	tuberous sclerosis 1	1.59	0.0005978	0.099
113	201926_s_at	CD55	CD55 molecule, decay accelerating factor for complement (Cromer blood group)	0.65	0.0005982	0.099
114	204070_at	RARRES3	retinoic acid receptor responder (tazarotene induced) 3	1.81	0.0006093	0.099
115	223210_at	CHURC1	churchill domain containing 1	1.35	0.00061	0.099
116	236921_at	EMB	embigin homolog (mouse)	3.49	0.0006115	0.099
117	203865_s_at	ADARB1	adenosine deaminase, RNA-specific, B1 (RED1 homolog rat)	0.45	0.00067	0.106
118	214508_x_at	CREM	cAMP responsive element modulator	0.30	0.0006719	0.106
119	218684_at	LRRC8D	leucine rich repeat containing 8 family, member D	1.72	0.0006737	0.106
120	208749_x_at	FLOT1	flotillin 1	0.34	0.0006873	0.106
121	228146_at	C17orf51	chromosome 17 open reading frame 51	0.55	0.0007012	0.106
122	229670_at	NA	NA	1.98	0.0007024	0.106
123	208997_s_at	UCP2	uncoupling protein 2 (mitochondrial, proton carrier)	1.23	0.0007152	0.106
124	213138_at	ARID5A	AT rich interactive domain 5A (MRF1-like)	0.35	0.000716	0.106
125	209488_s_at	RBPMS	RNA binding protein with multiple splicing	0.28	0.0007178	0.106
126	225457_s_at	LOC25845	hypothetical LOC25845	0.74	0.0007193	0.106
127	217999_s_at	PHLDA1	pleckstrin homology-like domain, family A, member 1	0.20	0.0007212	0.106
128	212310_at	MIA3	melanoma inhibitory activity family, member 3	1.50	0.0007293	0.106
129	224156_x_at	IL17RB	interleukin 17 receptor B	0.37	0.0007308	0.106
130	216060_s_at	DAAM1	dishevelled associated activator of morphogenesis 1	1.84	0.0007412	0.107
131	221059_s_at	CHST6	carbohydrate (N-acetylglucosamine 6-O) sulfotransferase 6	1.31	0.0007507	0.107
132	244035_at	BCL2	B-cell CLL/lymphoma 2	0.16	0.0007528	0.107
133	238295_at	C17orf42	chromosome 17 open reading frame 42	2.01	0.0007575	0.107
134	229021_at	NA	NA	0.70	0.000769	0.108
135	224686_x_at	LRRC37A2	leucine rich repeat containing 37, member A2	1.48	0.0007839	0.108
136	202932_at	YES1	v-yes-1 Yamaguchi sarcoma viral oncogene homolog 1	0.50	0.0007845	0.108

137	202377_at	NA	NA	0.63	0.0007875	0.108
138	230108_at	PGBD3	piggyBac transposable element derived 3	1.65	0.0008055	0.108
139	218352_at	RCBTB1	regulator of chromosome condensation (RCC1) and BTB (POZ) domain containing protein 1	1.27	0.0008082	0.108
140	201945_at	FURIN	furin (paired basic amino acid cleaving enzyme)	0.42	0.0008085	0.108
141	212741_at	MAOA	monoamine oxidase A	0.41	0.000813	0.108
142	218242_s_at	SUV420H1	suppressor of variegation 4-20 homolog 1 (Drosophila)	1.40	0.0008187	0.108
143	223217_s_at	NFKBIZ	nuclear factor of kappa light polypeptide gene enhancer in B-cells inhibitor, zeta	0.25	0.000844	0.110
144	215388_s_at	CFHR1	complement factor H-related 1	0.32	0.0008479	0.110
145	214030_at	DKFZp667G2110	hypothetical protein DKFZp667G2110	0.48	0.0008561	0.110
146	226981_at	MLL	myeloid/lymphoid or mixed-lineage leukemia (trithorax homolog, Drosophila)	1.59	0.000867	0.110
147	227701_at	C10orf118	chromosome 10 open reading frame 118	1.61	0.0008775	0.110
148	213766_x_at	GNA11	guanine nucleotide binding protein (G protein), alpha 11 (Gq class)	0.53	0.0008794	0.110
149	235424_at	NA	NA	1.70	0.0008808	0.110
150	207113_s_at	TNF	tumor necrosis factor (TNF superfamily, member 2)	0.09	0.0008842	0.110
151	217967_s_at	FAM129A	family with sequence similarity 129, member A	1.35	0.0008887	0.110
152	202149_at	NEDD9	neural precursor cell expressed, developmentally down-regulated 9	0.41	0.0008984	0.110
153	222907_x_at	TMEM50B	transmembrane protein 50B	1.74	0.0008997	0.110
154	1558747_at	SMCHD1	structural maintenance of chromosomes flexible hinge domain containing 1	1.12	0.0009019	0.110
155	214579_at	NPAL3	NIPA-like domain containing 3	1.35	0.0009054	0.110
156	1553117_a_at	STK38	serine/threonine kinase 38	2.03	0.0009219	0.110
157	226914_at	ARPC5L	actin related protein 2/3 complex, subunit 5-like	0.49	0.0009226	0.110
158	202859_x_at	IL8	interleukin 8	0.15	0.0009375	0.111
159	201417_at	SOX4	SRY (sex determining region Y)-box 4	3.49	0.0009393	0.111
160	226963_at	BTF3L4	basic transcription factor 3-like 4	0.74	0.0009503	0.111

161	207163_s_at	AKT1	v-akt murine thymoma viral oncogene homolog 1	0.67	0.0009578	0.112
162	219073_s_at	OSBPL10	oxysterol binding protein-like 10	0.52	0.0009665	0.112
163	204373_s_at	CEP350	centrosomal protein 350kDa	1.26	0.0009785	0.112
164	208892_s_at	DUSP6	dual specificity phosphatase 6	0.02	0.0009803	0.112
165	224989_at	NA	NA	2.47	0.0009863	0.112
166	210164_at	GZMB	granzyme B (granzyme 2, cytotoxic T-lymphocyte-associated serine esterase 1)	0.60	0.0009974	0.113

Genes selected at the nominal univariate comparison level of 0.001.
