# Supplementary material for: Molecular signature of cell cycle exit induced in human T lymphoblasts by IL-2 withdrawal
Source: BMC Genomics. 2009 Jun 8;10:261. doi: 10.1186/1471-2164-10-261 (PMC2706892; doi:10.1186/1471-2164-10-261)
Supplement: Additional File 3 — Table A3. Gene Ontology (GO) analysis of genes selected in a non-paired sample comparison between IL-2-deprived and non-deprived lymphocytes. The number of genes changed in each category was compared with the number of expected occurrences. Only GO classes and parent classes with at least 5 observations in the selected subset and with an "observed vs. expected" ratio of at least 2 were shown. [file 1471-2164-10-261-S3.pdf]

Table A3. Gene Ontology (GO) analysis of genes selected in a non-paired sample comparison between IL-2-deprived and non-deprived lymphocytes (see Additional file 1).

**Cellular Component**

| GO ID | GO Term | Observed in the selected subset | Expected in the selected subset | Observed/Expected |
|---|---|---|---|---|
| GO:0009986 | cell surface | 5 | 0.74 | 6.74 |
| GO:0005615 | extracellular space | 7 | 1.74 | 4.02 |
| GO:0005768 | endosome | 5 | 1.51 | 3.32 |
| GO:0044421 | extracellular region part | 8 | 2.92 | 2.74 |

**Molecular Function**

| GO ID | GO Term | Observed in the selected subset | Expected in the selected subset | Observed/Expected |
|---|---|---|---|---|
| GO:0016868 | intramolecular transferase activity, phosphotransferases | 5 | 0.11 | 43.89 |
| GO:0005126 | hematopoietin/interferon-class (D200-domain) cytokine receptor binding | 6 | 0.23 | 26.33 |
| GO:0016866 | intramolecular transferase activity | 5 | 0.25 | 19.75 |
| GO:0030246 | carbohydrate binding | 5 | 1.16 | 4.29 |
| GO:0016853 | isomerase activity | 7 | 2.13 | 3.29 |
| GO:0016881 | acid-amino acid ligase activity | 5 | 1.7 | 2.95 |
| GO:0019899 | enzyme binding | 6 | 2.33 | 2.58 |
| GO:0004721 | phosphoprotein phosphatase activity | 5 | 2.04 | 2.45 |

**Biological Process**

| GO ID | GO Term | Observed in the selected subset | Expected in the selected subset | Observed/Expected |
|---|---|---|---|---|
| GO:0007259 | JAK-STAT cascade | 8 | 0.48 | 16.68 |
| GO:0009968 | negative regulation of signal transduction | 6 | 0.88 | 6.84 |
| GO:0018193 | peptidyl-amino acid modification | 5 | 1.01 | 4.93 |
| GO:0001558 | regulation of cell growth | 5 | 1.07 | 4.68 |
| GO:0040008 | regulation of growth | 5 | 1.32 | 3.8 |
| GO:0016049 | cell growth | 5 | 1.38 | 3.61 |
| GO:0008361 | regulation of cell size | 5 | 1.38 | 3.61 |
| GO:0051674 | localization of cell | 9 | 2.88 | 3.13 |
| GO:0006928 | cell motility | 9 | 2.88 | 3.13 |
| GO:0007610 | behavior | 5 | 1.71 | 2.92 |
| GO:0040007 | growth | 5 | 1.82 | 2.74 |
| GO:0042221 | response to chemical stimulus | 9 | 3.44 | 2.62 |
| GO:0007267 | cell-cell signaling | 7 | 2.93 | 2.39 |

The number of genes changed in each category was compared with the number of expected occurrences. Only GO classes and parent classes with at least 5 observations in the selected subset and with an 'observed vs. expected' ratio of at least 2 were shown.
