# Supplementary material for: Molecular signature of cell cycle exit induced in human T lymphoblasts by IL-2 withdrawal
Source: BMC Genomics. 2009 Jun 8;10:261. doi: 10.1186/1471-2164-10-261 (PMC2706892; doi:10.1186/1471-2164-10-261)
Supplement: Additional File 4 — Table A4. Assessment of overall significance of expression changes following IL-2 deprivation in gene groups defined by Gene Ontology categories. Three independent tests: LS, KS and Efron-Tibshirani GSA were applied to select significantly affected gene classes. [file 1471-2164-10-261-S4.pdf]

Table A4. Assessment of overall significance of expression changes following IL-2 deprivation in gene groups defined by Gene Ontology (GO) categories.

GO category	GO ontology	GO term	Number of genes	LS permutation p-value	KS permutation p-value	Efron-Tibshirani's GSA test p-value
0004907	MF[1]	interleukin receptor activity	41	0.00001	0.00001	< 0.005
0019955	MF	cytokine binding	98	0.00001	0.00001	< 0.005
0019965	MF	interleukin binding	44	0.00001	0.0000949	< 0.005
0004896	MF	hematopoietin/interferon-class (D200-domain) cytokine receptor activity	68	0.00001	0.0000988	< 0.005
0007259	BP[2]	JAK-STAT cascade	52	0.00001	0.0001056	< 0.005
0006959	BP	humoral immune response	51	0.00001	0.0006364	< 0.005
0031060	BP	regulation of histone methylation	10	0.00001	0.0006694	< 0.005
0051568	BP	histone H3-K4 methylation	10	0.00001	0.0006694	< 0.005
0032844	BP	regulation of homeostatic process	7	0.00001	0.0015408	< 0.005
0032845	BP	negative regulation of homeostatic process	7	0.00001	0.0015408	< 0.005
0032847	BP	regulation of cellular pH reduction	7	0.00001	0.0015408	< 0.005
0048568	BP	embryonic organ development	22	0.00001	0.001863	< 0.005
0017153	MF	sodium:dicarboxylate symporter activity	9	0.00001	0.0037804	< 0.005
0005310	MF	dicarboxylic acid transmembrane transporter activity	9	0.00001	0.0037804	< 0.005
0006835	BP	dicarboxylic acid transport	9	0.00001	0.0037804	< 0.005
0050900	BP	leukocyte migration	24	0.00001	0.0047229	< 0.005
0017040	MF	ceramidase activity	10	0.00001	0.0049829	< 0.005
0009994	BP	oocyte differentiation	9	0.0001133	0.0003659	< 0.005
0048599	BP	oocyte development	9	0.0001133	0.0003659	< 0.005
0018212	BP	peptidyl-tyrosine modification	54	0.000114	0.002013	< 0.005
0051452	BP	cellular pH reduction	22	0.0001582	0.0040545	< 0.005
0051453	BP	regulation of cellular pH	22	0.0001582	0.0040545	< 0.005
0045851	BP	pH reduction	22	0.0001582	0.0040545	< 0.005
0051607	BP	defense response to virus	23	0.0001833	0.0030221	< 0.005
0018108	BP	peptidyl-tyrosine phosphorylation	53	0.0001871	0.0032075	< 0.005
0048477	BP	oogenesis	10	0.0003763	0.0033198	< 0.005
0006672	BP	ceramide metabolic process	43	0.0004208	0.0030228	< 0.005
0007260	BP	tyrosine phosphorylation of STAT protein	19	0.0004601	0.0046325	< 0.005
0001547	BP	antral ovarian follicle growth	8	0.0006383	0.0008385	< 0.005
0030218	BP	erythrocyte differentiation	45	0.0013289	0.00001	< 0.005
0042379	MF	chemokine receptor binding	32	0.001562	0.0008411	< 0.005
0006665	BP	sphingolipid metabolic process	65	0.001942	0.0026862	< 0.005

0001664	MF	G-protein-coupled receptor binding	37	0.0022013	0.0003398	< 0.005
0048660	BP	regulation of smooth muscle cell proliferation	6	0.003213	0.0021998	< 0.005
0048659	BP	smooth muscle cell proliferation	6	0.003213	0.0021998	< 0.005
0031543	MF	peptidyl-proline dioxygenase activity	6	0.0032605	0.0018536	< 0.005
0031545	MF	peptidyl-proline 4-dioxygenase activity	6	0.0032605	0.0018536	< 0.005
0019798	MF	procollagen-proline dioxygenase activity	6	0.0032605	0.0018536	< 0.005
0001541	BP	ovarian follicle development	15	0.0038025	0.0009442	< 0.005

Three independent tests, LS, KS and Efron-Tibshirani GSA, were applied to select significantly affected gene classes.

[1]MF – Molecular Function

[2]BP – Biological Process
