# Supplementary material for: Molecular signature of cell cycle exit induced in human T lymphoblasts by IL-2 withdrawal
Source: BMC Genomics. 2009 Jun 8;10:261. doi: 10.1186/1471-2164-10-261 (PMC2706892; doi:10.1186/1471-2164-10-261)

Figure A1. Hierarchical clustering analysis of selected memory T cell genes. The analysis based on the expression values of some of the genes identified by Holmes et. al [49] to characterise memory T cells. Samples before IL-2 withdrawal are in the light-blue columns, samples after IL-2 withdrawal in the navy-blue columns. Primary samples are in the yellow column, immortalised samples are in the green one.

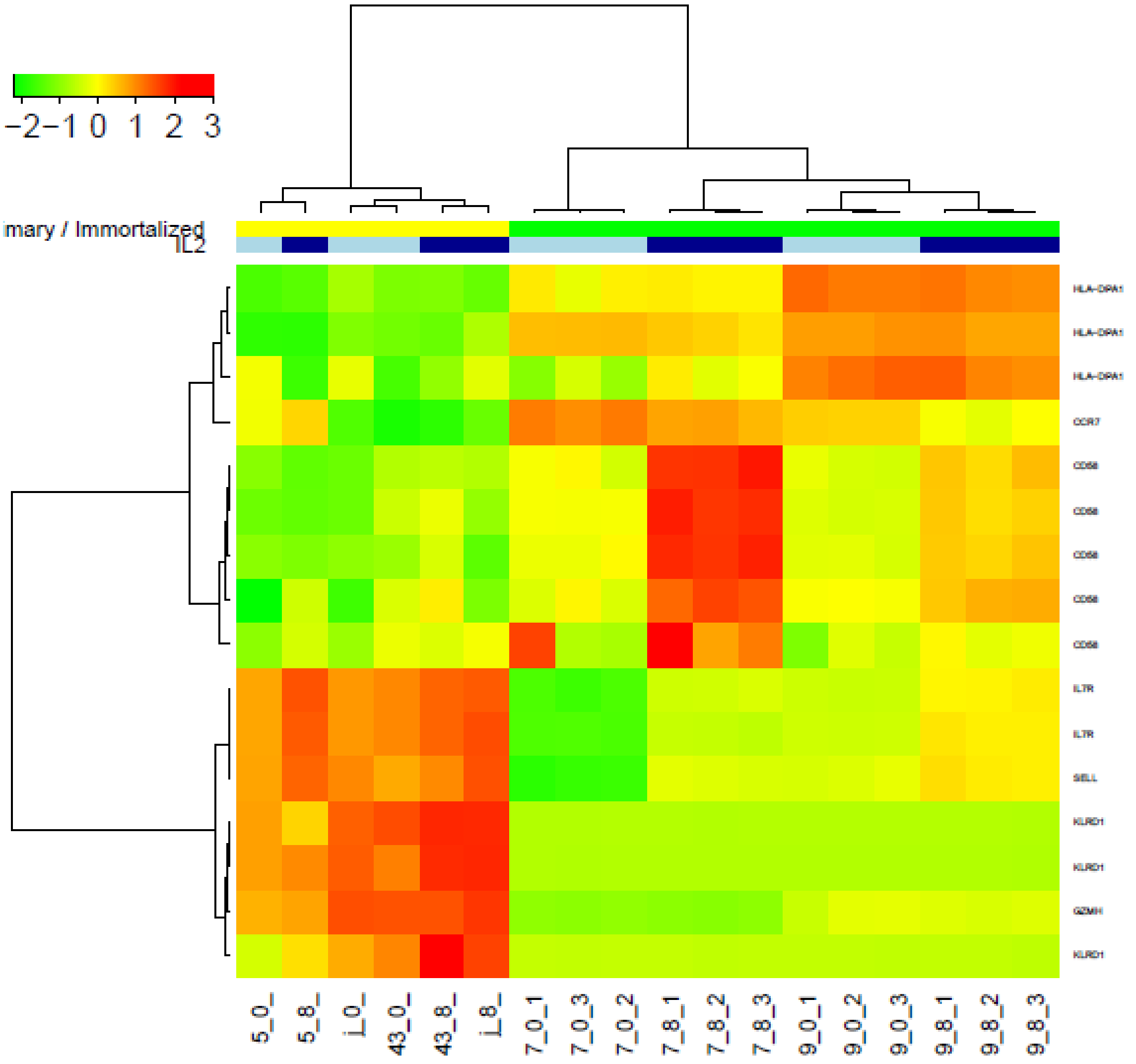

Supplement: Additional File 5 — Figure A1. Hierarchical clustering analysis of selected memory T cell genes. The analysis based on the expression values of some of the genes identified by Holmes et. al [49] to characterise memory T cells. Samples before IL-2 withdrawal are in the light-blue columns, samples after IL-2 withdrawal in the navy-blue columns. Primary samples are in the yellow column, immortalised samples are in the green one. [file 1471-2164-10-261-S5.pdf]
